# Genome-Wide Population Genetic Analysis of Commercial, Indigenous, Game, and Wild Chickens Using 600K SNP Microarray Data

**DOI:** 10.3389/fgene.2020.543294

**Published:** 2020-09-25

**Authors:** Jinxin Zhang, Changsheng Nie, Xinghua Li, Zhonghua Ning, Yu Chen, Yaxiong Jia, Jianlin Han, Liang Wang, Xueze Lv, Weifang Yang, Lujiang Qu

**Affiliations:** ^1^Department of Animal Genetics and Breeding, National Engineering Laboratory for Animal Breeding, College of Animal Science and Technology, China Agricultural University, Beijing, China; ^2^Beijing Municipal General Station of Animal Science, Beijing, China; ^3^Institute of Animal Sciences, Chinese Academy of Agricultural Sciences, Beijing, China

**Keywords:** chicken, genetic diversity, linkage disequilibrium, effective population size, Chinese indigenous chicken

## Abstract

Following chicken domestication, diversified chicken breeds were developed by both natural and artificial selection, which led to the accumulation of abundant genetic and phenotypic variations, making chickens an ideal genetic research model. To better understand the genetic structure of chicken breeds under different selection pressures, we genotyped various chicken populations with specific selection targets, including indigenous, commercial, gamecock, and wild ancestral chickens, using the 600K SNP array. We analyzed the population structure, genetic relationships, run of homozygosity (ROH), effective population number (Ne), and other genetic parameters. The wild ancestral population, red junglefowl (RJF), possessed the highest diversity, in comparison with all other domesticated populations, which was supported by linkage disequilibrium decay (LD), effective population number, and ROH analyses. The gamecock breeds, which were subjected to stronger male-biased selection for fighting-related traits, also presented higher variation than the commercial and indigenous breeds. Admixture analysis also indicated that game breed is a relatively independent branch of Chinese local breeds. Following intense selection for reproductive and productive traits, the commercial lines showed the least diversity. We also observed that the European local chickens had lower genetic variation than the Chinese local breeds, which could be attributed to the shorter history of the European breed. ROH were present in a breed specific manner and 191 ROH island were detected on four groups (commercial, local, game and wild chickens). These ROH islands were involved in egg production, growth and silky feathers and other traits. Moreover, we estimated the effective sex ratio of these breeds to demonstrate the change in the ratio of the two sexes. We found that commercial chickens had a greater sex imbalance between females and males. The commercial lines showed the highest female-to-male ratios. Interestingly, RJF comprised a greater proportion of males than females. Our results show the population genetics of chickens under selection pressures, and can aid in the development of better conservation strategies for different chicken breeds.

## Introduction

Abundant phenotypic and genotypic variations make the chicken an ideal model species for genetic studies. Following domestication in Southeast Asia (Liu et al., 2006; Storey et al., 2012; Miao et al., 2013), many breeds, including indigenous, commercial, and cockfighting chickens, were developed by artificial selection for different purposes. These diversified chicken breeds secured abundant genetic variants, such as various feather colors and comb types (Gunnarsson et al., 2007; Wright et al., 2009; Bed’hom et al., 2012; Dorshorst et al., 2015), which have played a pivotal role in the conservation and sustainable utilization of these genetic resources. Indigenous chickens were often subjected to both artificial and natural selection; however, they did not undergo strong selection. In comparison with local breeds, commercial chicken lines, mainly broilers and layers, have recently experienced a population bottleneck, resulting from intensified selection for meeting the demands of chicken eggs and meat. Owing to specific breeding targets, the selection of gamecock was biased to males to promote traits related to fighting, such as aggressiveness and stronger bones. During 1,000 years of genetic drift, and artificial and natural selection, most chicken breeds have adapted to their native environment and to human demands; thus, many genomic changes have occurred. The rich genetic diversity and extensive genetic basis of these breeds further provided excellent materials for heterosis and high-yield breeding (Tadano et al., 2007; Rubin et al., 2010). Population genetic analyses can help explain the history of these breeds, elucidate the origin and differentiation of breeds, and assist in genetic breeding. Such studies have already been conducted in many domesticated mammals and poultry, including chicken breeds, and cattle and pig populations (Maiorano et al., 2018; Zhang Z. et al., 2018; Elbeltagy et al., 2019).

In the poultry industry, most local breeds are seriously threatened by the impacts of commercial lines (Blackburn, 2006; Nie et al., 2019); only a few breeds, which match economic demands, are reared on a large scale. The use of a limited number of breeds is likely to cause a decrease in the genetic diversity of chickens, as commercial breeds and their hybrids are generated within a considerably shorter time to yield economic benefits. Indigenous chickens appear to be more genetically diverse than the commercial breeds, as they have been improved and established through a long breeding history, by processes remarkably different from those used for commercial breeds. Therefore, the conservation of local chicken breeds, as a genetic resource, is crucial to satisfy future unanticipated breeding demands (Chang et al., 2012; Khanyile et al., 2015).

Molecular tools provide geneticists an opportunity to estimate the genetic structure of different populations, using strategies such as genome-wide analyses, which have already been applied in many domestic animals (Johnsson et al., 2016; Zhang M. et al., 2018; Zhang Z. et al., 2018; Malomane et al., 2019). The popularity of single nucleotide polymorphism (SNP) analysis, such as microarrays and genomic sequencing, allows comprehensive evaluation of the population genetics of chickens (Khanyile et al., 2015; Nie et al., 2019). Therefore, in this study, we first assessed the population structure and diversity of various chicken breeds driven by different selection targets using 600K SNP microarray data and compared them with their wild ancestor, the red junglefowl (RJF). We also conducted run of homozygosity (ROH) detection and estimated the inbreeding coefficient of chicken breeds under different selection pressures. In addition, we calculated linkage disequilibrium (LD) decay, LD-based effective population sizes, and the effective sex ratio (ESR) of 15 breeds, to provide basic information for their management and conservation purposes.

## Materials and Methods

### Sample Collection and SNP Genotyping

**FIGURE 1:**
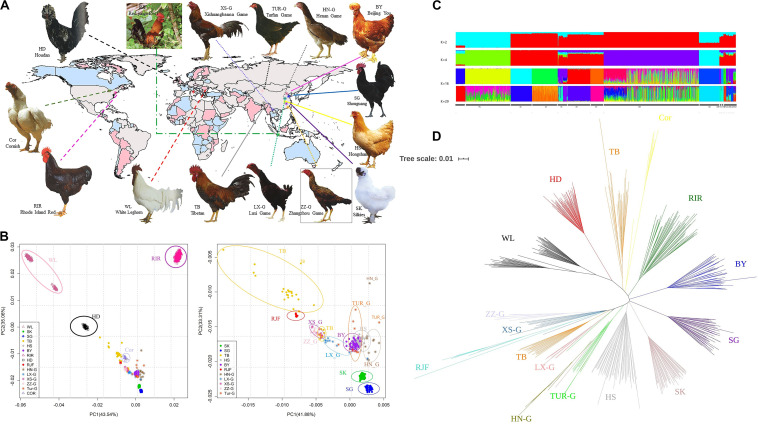
**(A)** Chicken breeds with distinct phenotypes and geographical information (Photos came from poultry genetic resources in china). **(B)** Principal component analysis revealing genetic differentiation of 15 populations using SNP data. Breeds which are labeled, their names are mentioned in the main text; The Commercial breeds are denoted by triangle symbols, The Chinese indigenous breeds are displayed as dots, RJFs are displayed as plus sign, Europe local breed is denoted by squares, and the rest populations are marked as asterisks; PCA was performed on the whole population, plotting the first and the second dimension revealed PCA of 15 populations. **(C)** The admixture plot for breeds analyzed based on different number of assumed ancestors (K). **(D)** Neighbor-joining tree constructed using PHYLIP.

In total, 1,254 chickens were randomly selected from 15 chicken populations, including five Chinese local breeds (Beijing You [BY], *n* = 56; Hongshan [HS], *n* = 96; Shouguang [SG], *n* = 109; Silkie [SK], *n* = 89; Tibet [TB], *n* = 41), one European local breed (Houdan [HD], *n* = 86), three commercial breeds (Rhode Island Red [RIR], *n* = 478; White Leghorn [WL], *n* = 230; Cornish [Cor], *n* = 10), five gamecock breeds (Luxi Game [LX-G], *n* = 10; Henan Game [HN-G], *n* = 11; Xishuangbanna Game [XS-G], *n* = 10; Zhangzhou Game [ZZ-G], *n* = 11; Turfan Game [Tur-G], *n* = 10), and the wild ancestral population (RJF, *n* = 7). Detailed information for these samples is presented in Table 1. The geographical distribution and photographs of the chickens included in our study are shown in Figure 1A.

**TABLE 1:** Categories of chicken breeds.

Population	Geographic Origin	Classification	Number
Silkies (SK)	Jiangxi Province, China	Indigenous Breeds	89
Shouguang (SG)	Shandong Province, China		109
Tibetan (TB)	Tibet, China		41
Hongshan (HS)	Hubei Province, China		96
Beijing You (BY)	Beijing, China		56
Houdan (HD)	France		86
Luxi Game (LX-G)	Shandong Province, China	Fight Breeds	10
Henan Game (HN-G)	Henan Province, China		11
Xishuangbanna Game (XS-G)	Yunnan Province, China		10
Zhangzhou Game (ZZ-G)	Fujian Province, China		11
Turpan Game (Tur-G)	Xinjiang, China		10
Rhode Island Red (RIR)	Rhode Island, America	Commercial Breeds	478
White Leghorn (WL)	Tuscany, Italy		230
Cornish (Cor)	Cornwall, England		10
Red jungle fowl (RJF)	Daerah Istimewa Aceh, Indonesia	Wild Breed	7
Total			1,254

*TB: data of 15 Samples were collected from the Experimental Chicken Farm at the China Agricultural University, 10 samples were collected form Nimu, Tibet, 13 Samples were collected form Naidong, Tibet and 3 samples were collected form Lingzhi, Tibet. WL: data of 39 were collected from the experimental Chicken Farm at China Agricultural University and 191 samples were collected form a commercial company in Yanqing, Beijing. RIR: data came from two batches, the first data has 96 samples, the second data has 382 samples.*

Genomic DNA was extracted from a blood sample using the TIANamp Blood DNA Kit DP348 (Tiangen Biotech Co., Ltd., Beijing, China). Genotyping was performed using the 600K Affymetrix Axiom Chicken Genotyping Array (Affymetrix, Inc., Santa Clara, CA, United States), and included 580,841 SNPs across the entire chicken genome (chromosome: 554316, Sexual-chromosome: 26525). Based on these data, SNPs were excluded from the analysis if the minor allele frequency and call rate were less than 0.05 and 0.90, respectively, and if the genotype frequencies deviated severely from the Hardy-Weinberg equilibrium, with a of *P*-value less than 10^–6^. After quality control, all genotyped individuals were retained for further analyses, SNP distribution is shown in Supplementary Table S1.

### Population Structure Analysis

Principal component analysis (PCA) was performed on all individuals using a GCTA (Yang et al., 2011). Two principal components and their linear discriminants were extracted as horizontal and vertical coordinates and visualized in R. A neighbor-joining (NJ) tree was constructed using the phylogeny program PHYLIP (Plotree and Plotgram, 1989) and displayed with iTOL (Letunic and Bork, 2011). The genetic structure of the 15 populations was analyzed with ADMIXTURE (Alexander et al., 2009), Thirty runs were performed, with *K* values ranging from 1 to 30. *K*-value (*K* = 29) had the lowest cross validation error (CV-error). The results for *K* = 2, *K* = 4, *K* = 16, and *K* = 29 are included in this report. The other *K*-value results and cross validation error plot of admixture are provided in Supplementary Figures S1, S2.

### Genetic Differentiation

Based on quality-controlled data, we calculated the pairwise *F*_ST_ (Weir and Cockerham, 1984) in 15 populations using VCFtools (Danecek et al., 2011). The *F*_ST_ value ranges from 0 to 1, where 0 indicates that the population is not differentiated, and 1 indicates that the population is completely differentiated. Genetic differentiation was divided into four levels according to the *F*_ST_ value: low (<0.05), medium (0.05–0.15), high (0.15–0.25), and extreme (>0.25) (Hartl et al., 1980).

### Linkage Disequilibrium Decay

*R*^2^ was used as a measure of LD in this study (VanLiere and Rosenberg, 2008). For the two SNP markers, the alleles were A, a, B, and b, and the corresponding allele frequencies were *P*_A_, *P*_a_, *P*_B_, and *P*_b_. The four haplotype frequencies formed between the alleles were *P*_AB_, *P*_ab_, *P*_aB_, and *P*_Ab_. Hence, the formula for the two markers was:

$$R^{2}=\frac{{\left(P_{\text{AB}}\,P_{\text{ab}}-P_{\text{Ab}}\,P_{\text{aB}}\right)}^{2}}{P_{\text{A}}\,P_{\text{a}}\,P_{B}\,P_{\text{b}}}$$

Seven individuals were randomly selected from each population, and PopLDdecay (Zhang et al., 2019) was used to measure the *R*^2^ of each population. In order to observe more intuitively the trend of LD, we used *R*^2^ as the ordinate, created a scatterplot with the physical distance between the paired SNPs (less than 500 kb) as the abscissa, and generated the curves using R.

### Effective Population Size

Effective population size (Ne) is a genetic population parameter that aids the understanding of the evolutionary history of populations and the genetic mechanisms underlying complex traits. For each breed, we calculated the Ne prevalence 1,000 generations ago, using SNeP (Barbato et al., 2015), to summarize the extent of LD. Contemporary Ne was further estimated according to the random mating model based on LD, using default parameters in NeESTIMATOR (version 2.01) (Do et al., 2014).

### Effective Sex Ratio

Sex bias is an important piece of information on demographic dynamics and social structure. The ESR refers to the proportion of females in the effective population. We calculated the ESR of the 15 populations based on a hierarchical Bayesian model, using KIMTREE (version 2.1) (Gautier and Vitalis, 2013; Clemente et al., 2018). To obtain accurate results, we generated 50 pseudo-replicated datasets by randomly selecting 5,000 autosomal and 5,000 sex-linked SNPs. The algorithm was started with 20 pilot runs of 500 iterations each to adjust the parameters of the Monte Carlo Markov Chain (MCMC). The MCMC itself was run for 20,000 generations and sampled every 20 iterations after a burn-in of 10,000 iterations.

This method analyzes the allele frequencies along each branch, using Kimura’s time-dependent diffusion approximation for genetic drift (Kimura, 1964). This considers the allele frequencies of sex chromosome linkage markers and autosomal markers to estimate the contribution of gene pools in males and females during the same period. Briefly, the ESR can be considered as a comparison analysis of the effective population sizes estimated from autosomes and the sex chromosome. The branch length (_τ_) is measured on a diffusion time scale, and it is proportional to the time since divergence in generations (t) scaled by the effective population sizes (Ne).

τ(A)≡t(2Ne(A))

τ(X)≡t(2Ne(X))

From the above formula, the ESR of our sample can be calculated from:

$$\varepsilon =2-\frac{9}{8}\,\frac{\tau^{\left(\text{X}\right)}}{\tau^{\left(\text{A}\right)}}$$

### Heterozygosity and Runs of Homozygosity

We used different parameters to characterize genetic diversity, all of which were obtained from PLINK (version 1.9) (Purcell et al., 2007; Chang et al., 2015). Genome-wide nucleotide diversity (π) was estimated for each population, using VCFtools v0.1.14 (Danecek et al., 2011) with default parameters.

The proportion of observed heterozygosity (Ho) was estimated from the observed homozygosity (-het) as 1–(the number of observed homozygous loci/number of non-missing loci), and the expected heterozygosity (He) was calculated as the 1–(the number of expected homozygous loci/number of non-missing loci). The expected heterozygosity and observed heterozygosity estimate for all individuals within each population were averaged over all SNPs.

The estimation of the inbreeding coefficient of individuals was based on the ROH. Long homozygous fragments were scanned using PLINK (version 1.9). Specific parameters were as follows: a sliding window of 50 SNP slides along the chromosome to estimate homozygosity; each sliding window allowed no more than one heterozygote, with no more than five missing SNPs, a minimum length of ROH of 100 kb, a minimum density of 1 SNP/50 kb, and a maximum gap between consecutive SNPs of 1,000 kb.

To identify the genomic regions that were most commonly associated with ROH in different chicken breeds, we estimated the percentage of occurrences of SNPs in ROH by counting the number of times when the SNP was detected in those ROH. The genomic regions most often associated with ROHs were identified by selecting the top 1% SNPs observed in ROHs. Adjacent SNPs over this threshold were merged into genomic regions named ROH islands (Purfield et al., 2017; Mastrangelo et al., 2018). The ROH islands were annotated using the Ensembl BioMart (Smedley et al., 2009).

Inbreeding coefficients (F_ROH_) for each breed were calculated according to McQuillan (McQuillan et al., 2008), using the following formula:

$${\text{F}}_{\text{ROH}}=\sum \frac{{\text{L}}_{\text{ROH}}}{{\text{L}}_{\text{AUOT}}}$$

where L_AUTO_ is the length of the autosomal genome spanning the SNP positions (952,090 kb in the present study).

## Results

### Population Structure Analysis

Population structures of the 15 breeds, comprising four commercial breeds, 10 Chinese indigenous breeds, and one wild breed, were analyzed using PCA, NJ tree, and ADMIXTURE. The PCA showed that the top two principal components accounted for 43.54% (PC1) and 35.06% (PC2) of the total variability (Figure 1B). For each population, all individuals were grouped together, showing a consistent genetic relationship. WL from the two sites was clearly grouped into the respective clusters (Table 1). To further understand the genetic structure of indigenous breeds in China, we removed the four imported lines and re-performed the PCA (Figure 1B). The results showed that RJF, Tibetan, Shouguang, and Silkies were further away from other breeds, which may be associated with their geographic location, selection targets, and production performance, among other factors. The Tibetan Silkies were found at four sites clustered more loosely with each other, indicating greater genetic variation within the breed. We also observed that the five fighting chicken breeds were similar in their genetic structure, indicating potential genetic admixture among them.

Admixture analysis results of all sampled chickens were generated using a model-based clustering approach by ADMIXTUREV (version 1.3) (Figure 1C and Supplementary Figure S1). At *K* = 2, WL was separated from the other breeds. The genetic backgrounds of WL and other breeds are clearly significantly different. At *K* = 4, commercial breeds, except for Cornish, were identifiable from indigenous breeds. The Cornish breed contains fighting chicken blood, so it was separated later. At *K* = 8 and *K* = 11, Beijing You and Tibet were separated. Cornish, RJF and game breeds always grouped into one population, until *K* = 16. The optimal *K*-value for admixture was *K* = 29. All breeds exhibited independent genetic background, except for game chickens. The distinction between different populations of chicken game is blurred. The NJ tree (Figure 1D) was consistent with the PCA and admixture results; game chickens, Chinese local chickens, foreign chickens, and RJF were clearly distinguishable from each other.

### Genetic Differentiation Analysis

**FIGURE 2:**
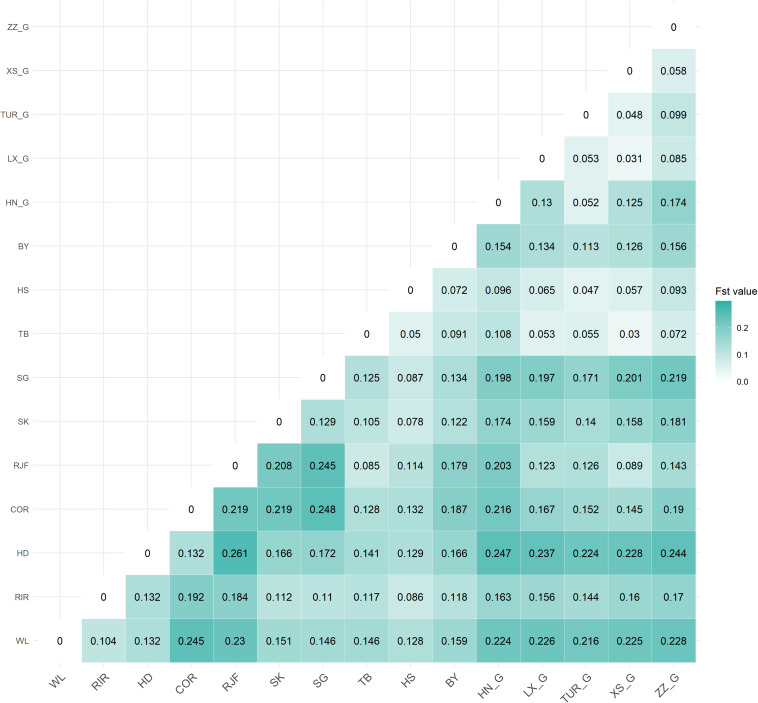
Pairwise estimates of genetic differentiation (F_ST_) between fifteen populations.

The genetic differentiation (*F*_ST_ values) pairwise estimates between 15 chicken populations are shown in Figure 2. Genetic differentiation had occurred among different varieties. The average *F*_ST_ values ranged from 0.03 to 0.27. First, the *F*_ST_ of commercial breeds and *F*_ST_ of Chinese indigenous breeds had the highest differentiation, especially Cornish. The wild breed (RJF) and *F*_ST_ value of Tibetan chickens was 0.08, indicating less differentiation than other breeds. This result was confirmed by PCA and NJ analyses, which supported that Tibetan chicken was adjacent to RJF. Finally, there was greater differentiation between indigenous chickens and fighting chickens (0.06–0.25), but the degree of differentiation between game chicken subgroups was lower (0.03–0.17).

### Heterozygosity and Runs of Homozygosity

The proportion of nucleotide diversity (π) and mean value of expected heterozygosity (He) and observed heterozygosity (Ho) for each population are shown in Table 2. Overall, the diversity of most local chicken breeds was higher than that of the commercial breeds. Of these, the expected heterozygosity of RJF (π = 0.34) and Luxi Game (π = 0.35) populations were the highest, followed by the local breeds and commercial breeds (RIR:0.30, WL:0.32). Among the commercial lines, compared with layer chickens (WL, RIR), broiler chickens (Cornish) were more diverse (He = 0.35, π = 0.36) and at almost the same level as the wild ancestral chickens. This may be related to the broader genetic basis of founder populations and more crossbreeding.

**TABLE 2:** The genetic diversity estimates for different type breeds.

Population	Ho^1^	Range	He^2^	Range	π^3^	Ne^4^
White Leghorn	0.29 ± 0.02	(0.35,0.23)	0.30 ± 0.00	(0.30,0.30)	0.30 ± 0.16	109
Rhode Island Red	0.32 ± 0.02	(0.43,0.28)	0.32 ± 0.00	(0.32,0.32)	0.33 ± 0.15	189
Cornish	0.39 ± 0.04	(0.45,0.35)	0.35 ± 0.00	(0.34,0.34)	0.36 ± 0.14	31
Red jungle fowl	0.29 ± 0.05	(0.36,0.20)	0.34 ± 0.00	(0.34,0.34)	0.37 ± 0.14	15
Houdan	0.29 ± 0.01	(0.33,0.27)	0.30 ± 0.00	(0.30,0.30)	0.30 ± 0.16	153
Beijing You	0.33 ± 0.01	(0.35,0.28)	0.33 ± 0.00	(0.33,0.33)	0.34 ± 0.15	59
Silkies	0.34 ± 0.01	(0.40,0.31)	0.33 ± 0.00	(0.33,0.33)	0.33 ± 0.15	134
Shouguang	0.34 ± 0.01	(0.36,0.30)	0.34 ± 0.00	(0.34,0.34)	0.33 ± 0.15	100
Tibetan	0.29 ± 0.02	(0.36,0.20)	0.32 ± 0.00	(0.32,0.32)	0.33 ± 0.15	88
Hongshan	0.33 ± 0.01	(0.34,0.27)	0.33 ± 0.00	(0.33,0.33)	0.33 ± 0.15	140
Henan Game	0.32 ± 0.08	(0.47,0.21)	0.29 ± 0.00	(0.29,0.29)	0.31 ± 0.16	18
Luxi Game	0.36 ± 0.03	(0.39,0.28)	0.35 ± 0.00	(0.35,0.35)	0.37 ± 0.14	20
Turfan Game	0.33 ± 0.04	(0.42, 0.27)	0.33 ± 0.00	(0.33,0.33)	0.35 ± 0.15	50
Xishuangbanna Game	0.34 ± 0.02	(0.31,0.36)	0.34 ± 0.00	(0.34,0.35)	0.36 ± 0.14	22
Zhangzhou Game	0.36 ± 0.01	(0.34,0.39)	0.34 ± 0.00	(0.34,0.34)	0.36 ± 0.15	20

*^1^Ho = Observed Heterozygosity. ^2^He = Expected Heterozygosity. ^3^π = Genome-wide nucleotide diversity. ^4^Ne = Effective population size.*

Runs of homozygosity can reflect the degree of animal inbreeding. A long Runs of homozygosity means inbred animals with recent common ancestors, whereas a short Runs of homozygosity reflects more distant common ancestors. We summarized the runs of homozygosity and genomic inbreeding coefficient (F_ROH_) of 15 chicken breeds (Table 3). The number, length, and inbreeding coefficient of ROH per chicken population were significantly different. The average total length of runs of homozygosity varies from 76,825.71 kb (RJF) to 280,261.03 kb (Houdan). A comparison across the 15 populations showed that commercial breeds had longer runs of homozygosity, suggesting that recent inbreeding had occurred in these populations. The individual *F*_ROH_ values varied from 0.048 to 0.35. The average F_ROH_ of three commercial breeds and foreign indigenous breed (Houdan) exceeded 0.22, whereas Chinese indigenous breeds, game breeds, and RJF had lower inbreeding levels. Our results suggested that there were clear variations in the inbreeding of ROH arising from the selection pressure experienced by the chickens.

**TABLE 3:** Statistical for runs of homozygosity and genomic inbreeding coefficient in different type breeds.

Population	NSEG^1^	KB (kb)^2^	F_ROH_^3^	Range
White Leghorn	94.6415.03	267176.1457046.71	0.28 ± 0.06	(0.44,0.02)
Rhode Island Red	64.8614.89	182716.8160865.74	0.22 ± 0.05	(0.32,0.02)
Cornish	63.912.53	227566.0046589.86	0.24 ± 0.08	(0.36,0.14)
Houdan	89.947.09	280261.0325679.66	0.35 ± 0.03	(0.21,0.03)
Beijing You	47.166.18	136076.0025274.47	0.20 ± 0.04	(0.42,0.27)
Silkies	43.526.38	128013.1025912.37	0.17 ± 0.03	(0.32,0.11)
Shouguang	61.325.89	182585.1324056.17	0.19 ± 0.03	(0.22,0.04)
Tibetan	25.7810.21	87618.0546779.5	0.16 ± 0.06	(0.27,0.14)
Hongshan	11.34.70	40147.2231312.72	0.08 ± 0.05	(0.42,0.02)
Red jungle fowl	34.7114.77	76825.7135958.9	0.08 ± 0.06	(0.27,0.01)
Henan Game	49.3624.92	203347.52136431.7	0.23 ± 0.17	(0.50,0.01)
Luxi Game	14.516.48	30699.6339080.21	0.09 ± 0.05	(0.20,0.02)
Turfan Game	26.0915.20	81137.1962520.44	0.12 ± 0.06	(0.19,0.002)
Xishuangbanna Game	8.85.65	37234.2537676.86	0.05 ± 0.04	(0.14,0.004)
Zhangzhou Game	3710.22	96595.1833172.07	0.11 ± 0.04	(0.18,0.04)

*^1^NSEG = Number of runs of homozygosity. ^2^KB = Total length of runs (kb). ^3^F_*ROH*_ = Inbreeding Coefficient.*

**FIGURE 3:**
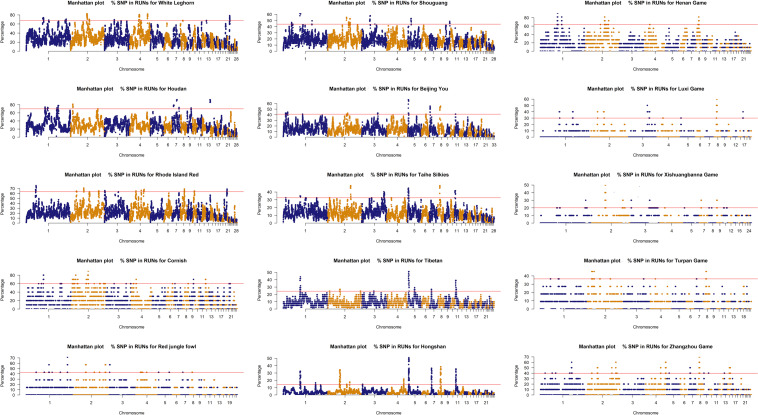
Manhattan plot of occurrences (%) of a SNP in the runs of homozygosity for per breed.

The ROH islands were identified in the 15 chicken breeds, and the percentage of SNPs in ROH was plotted against the positions of the SNPs along the chromosomes in Figure 3. For per breed, the percent of the autosome residing in a ROH was not uniformly distributed. ROH islands threshold (top 1% SNPs) was ranged from 14 to 69% in chicken breeds, suggesting that occurrence of ROH in breed under unequal selection pressures was significantly different. Commercial breed was over 60%, local, game and wild breeds were 14–44, 20–63, and 43%, respectively. A total of 191 regions were detected as genomic regions with a high frequency of ROH among which 50 regions were commercial breeds, 18 regions were wild breed, 43 regions were game breeds 51 regions were local breeds and 29 regions did not contain genes, covered 1,598 genes (Supplementary Table S2).

### Estimation of Genome-Wide Linkage Disequilibrium and Effective Population Size

**FIGURE 4:**
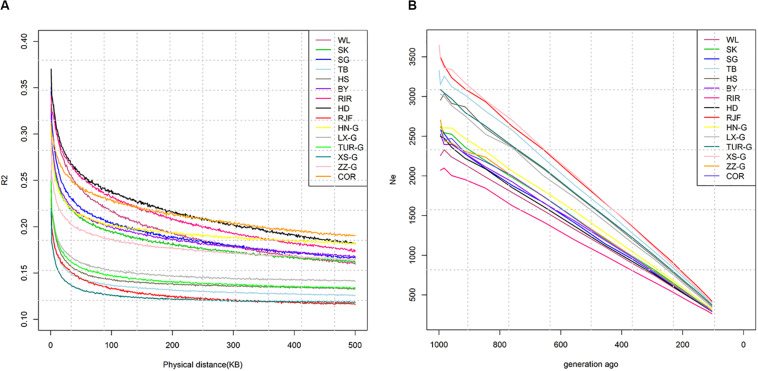
**(A)** Decay in linkage disequilibrium (r^2^) over increasing intermarker distance in different population. **(B)** The trend in modern effective population size of different breeds.

Whole-genome-wide LD of all populations were analyzed using genomic data from high-density SNP chips. The extent of LD was first evaluated for all pairwise allele combinations of marker pairs. PopLDdecay was used to calculate the LD, and sites with marker distances less than 500 kb were selected to develop the LD decay curves. According to the principle of selecting the *R*^2^ value at 5,000 bps, the sample means of these *R*^2^ values were calculated, and the genome-wide LD statistics of the 15 populations were plotted. The results are shown in Figure 4A. As the physical distance between the markers increased, the LD between the markers decreased, and the degree of LD attenuation differed between the different populations. The commercial lines, which have been subjected to long-term intensive selection, exhibited a slower LD decay than wild chickens and other local breeds. For distances less than 150 kb in the commercial breeds, LD was the highest in RIR, followed by Cornish and WL. For distances greater than 150 kb, LD was the highest in Cornish, followed by RIR and WL. LD decreased steadily in the local breeds, and it was lower in Chinese local breeds than in the European local breed (Houdan), and in the wild breeds than in the fighting breeds. RJF exhibited the fastest LD decay and the smallest R^2^, indicating the higher diversity of its wild ancestors.

The average values of effective population size over the past 1,000 generations estimated from the mean *R*^2^ values of the 15 chicken breeds/populations, using the default Minor Allele Frequency (0.05) thresholds in SNeP, are shown in Figure 4B. A continuous decline in effective population size (Ne) was found across generations within the analyzed period. The wild-type RJF and commercial RIR had the largest and smallest effective population size, respectively. The effective population size of the local and gamecock breeds was intermediate. WL, RIR, and Cornish are well-known commercial breeds, and the size of Ne was congruent with their breeding history. Owing to the differences in populations, the Ne of each breed was different; however, all effective population size showed a downward trend in recent years.

In addition, 40 individuals were randomly selected from each group to calculate contemporary Ne based on LD (seven individuals of RJF). The results are shown in Table 2. The average Ne was noticeably different from the modern Ne for all 15 populations (ranging from 15 to 189).

### Estimation of Effective Sex Ratio

**FIGURE 5:**
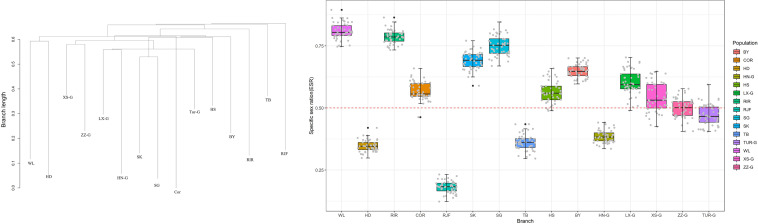
The boxplots summarize the distributions of the posterior mans of the ESR for each population, for the 50 pseudo-replicated datasets. The dotted line indicates a balanced ESR (ξ = 0.5).

Effective sex ratio is critical for a better understanding of social structures and demographic dynamics. We used KIMTREE (Gautier and Vitalis, 2013; Clemente et al., 2018) to infer the ESR of the populations. The results showed that the ESR of different populations was significantly different. Furthermore, it was evident that the non-commercial breeds, such as Houdan, Tibetan, RJF, and Henan Game, showed a strongly male-biased ESR, whereas a female-biased ratio was observed in commercial breeds. Commercial breeding patterns, such as those involving artificial insemination, may contribute to a significant increase in the effective proportion of females in the population. The RJF group exhibited the strongest bias, with a female-to-male ratio of approximately 1:5, suggesting strong sex bias in the population or strong male-biased dispersal during population expansion (Figure 5).

## Discussion

### Population Structure and Genetic Diversity

We assessed the population structure of 15 different chicken breeds and showed that genetic background significantly differed among them. Based on PCA and NJ tree analysis, individuals of each population clustered together and exhibited a consistent genetic composition. The exotic breeds were clearly separated from the Chinese local breeds. However, Tibetan and RJF exhibited a close relationship, which agrees with the result claimed by Bao (Bao et al., 2008). There are two explanations for this: Tibetan chickens are found on the Qinghai-Tibet plateau, in a relatively isolated environment, are often bred with little or no selection and admixture results showed that Tibetan chicken shared similar genetic background with RJF. In addition, game chickens are a group of ancient breeds that have been bred for the purpose of cock fighting. To prevent the reduction of aggression, which leads to the loss of breed characteristics, the breeding of game chickens often avoids crossbreeding with other type chickens, so it is a relatively independent branch of Chinese local breeds. They possess their own genetic structure in subgroups. In admixture result, game breeds and red jungle clustered as one group until *K* = 16, suggesting domestic breeds and wild populations have mutual introgression (Li et al., 2019; Wang et al., 2020). It is consistent with our NJ tree results that all breeds from the same population clustered, except that game breeds. Luxi Game, Henan Game and Turfan Game mixed together.

Based on the *F*_ST_ values, game chicken is more distant from other Chinese local breeds (0.05–0.22), and their subpopulation genetic differentiation is smaller (0.03–0.17). However, Zhangzhou Game has been mixed with foreign chicken blood (Zhu et al., 2009). Thus, there was vast population differentiation between the Zhangzhou Game and four other native game breeds. This result was consistent with the research reported by Qu et al. (2006) and Zhu et al. (2009).

China has an abundance of indigenous chicken breeds with diverse traits. Our results indicated that those breeds have higher genetic diversity (heterozygosity and nucleotide diversity) than exotic commercial breeds, which is in agreement with previous studies (Chen et al., 2018; Nie et al., 2019). For local breeds, observed heterozygosity (Ho) was either equal to or higher than expected heterozygosity (He), indicating that diversity protection has improved in recent years, as previously reported (Zhang M. et al., 2018). Game chickens are mostly used in competitive games, and crossbreeding is quite extensive among them, which has resulted in heterosis and greater genetic diversity (Liu and Zhao, 2010). Previous studies have shown that the genetic diversity of commercial lines was reduced by nearly 50% (Muir et al., 2008). In our study, low heterozygosity and polymorphism were observed in layer chickens. Strong and standardized production causes the loss of individual characteristics and results in a low level of diversity. It may affect the flexibility to respond unforeseen future needs. In production, the intensity of selection of chickens should be controlled while taking into account the production performance.

Red junglefowl, which is considered to be the ancestor of the domesticated chicken (Murray, 2016; Wang et al., 2020), showed no significant difference in the heterozygosity diversity between the Chinese local chicken breeds in our study. Malomane et al. (2019) has reported similar observed heterozygosity in RJF compared with that of our study. However, we found that the standard deviation of the heterozygosity of Red jungle fowl was greater than that of other breeds. This suggests that the diversity of Red jungle fowl exhibited a wide range of fluctuations. Owing to the influence of human activities, the distribution and population size of wild RJF have decreased, which has led to a decline in their genetic diversity. The main threats to RJF populations were reported to be egg harvesting and hunting (Akrim et al., 2015; Setianto et al., 2017). Hence, the protection of RJF should not be ignored.

### Genomic Inbreeding and Runs of Homozygosity Island

In general, there are differences in the length of ROH in the genome of chickens in various production directions. Commercial breeds had the largest Runs of homozygosity (183–280 Mb), followed by local and wild breeds (37–183 Mb; 76 Mb). This reflects the difference in the degree of inbreeding and the population history. The higher the inbreeding level, the greater the Runs of homozygosity number and length in the genome. According to our results, WL, RIR and Cornish might experience strong inbreeding. There are several likely reasons for this: first, strict breed standards causing breeders use inbreeding to select outstanding offspring; second, the import number limit due to frequent use of a few male chickens. The utility of Runs of homozygosity to estimate inbreeding has already been proven in cattle (Kim et al., 2013; Zhang et al., 2015), pigs (Bosse et al., 2015), and goats (Onzima et al., 2018). The heterozygosity of the selected region was reduced and the homozygosity increased (Smith and Haigh, 1974).

Continuous directional selection was more likely to generate high frequency ROH regions. In our study, a large number of ROH regions were identified. The ROH islands contained many genes potentially affecting breed specific traits (Supplementary Table S2). We identified several candidate genes related to breed diversity. On chromosome 11, more than 33% of the Silkies chickens, 24% of the Tibet chickens and 14% of Hongshan chickens had a ROH in the region from 1.91 to 4.00 Mb, from 2.31 to 5.66 Mb, and from 2.30 to 3.91 Mb, which contained *NECAB2*, *SLC38A8*, and *NFATC3* genes, and several potential candidate genes for silky feathers. On chromosome 3, we showed that up to 40% of Beijing You chickens had a ROH between 69.38 and 70.93 Mb, retaining the gene *ASCC3* which is also a candidate gene for silky feathers (Li et al., 2019). Besides, on chromosome 2, chromosome 3, and chromosome 13, we identified several potential candidate genes associated with egg production, eggshell quality and growth traits, such as *GDF9*, *OPN*, *CDH9*, *CDH10*, and *KIF18A* (Pines et al., 1995; Hincke et al., 2010; An et al., 2012; Lin et al., 2014; Wang et al., 2020). The ROH island results tended to provide useful information on genetic diversity and genetic foundation underpinning breed specific traits under different selection pressures.

### Linkage Disequilibrium and Effective Population Size Analyses

We investigated LD decay and the effective population size using 15 different breeds (including Chinese local breeds, commercial breeds, and wild breeds). Our results showed that LD decreased with an increase in the physical distance between SNPs, and that indigenous breeds displayed lower LD than commercial chickens, which was consistent with the results of previous studies (Chen et al., 2018; Nie et al., 2019). Previous research has confirmed that crossbreds shown lower LD than inbred populations (Karlsson et al., 2007; Seo et al., 2018; Nie et al., 2019), and that LD increased with the inbreeding rate and decreased with an increase in hybridization (Aerts et al., 2007). Their results were consistent with ours. For the example of game breeds: to increase fighting power, crossbreeding is quite extensive among game chickens; hence, the game chickens displayed lower LD value than other local breeds. Among them, Henan Game had the highest LD value and inbreeding coefficient (F_ROH_: 0.23). Xishuangbanna Game had the lowest LD value and inbreeding coefficient (F_ROH_: 0.05). The other breeds followed the same trend. The effective population sizes were opposite to their LD level.

We used whole-genome SNP data to estimate effective population size. The results showed that the effective population size of all populations decreased over time, which may have been caused by a bottleneck effect, variety differentiation, or selection pressure during domestication (Figure 4B). The wild-type RJF exhibited the fastest decline rate (Figure 4B), and the decline rate of local breeds was higher than that of commercial chickens. Other studies have also shown that the effective population of local breeds is shrinking (Khanyile et al., 2015). A reduction in the effective population will affect genetic progress.

### Unequal Diversity Between Sex and Autosomal Chromosomes

The proportion of females in the effective population, known as the ESR, can provide information about the reproductive potential of a population, which is essential for wildlife management of endangered species, and to better understand the underlying social structure and population dynamics (Morrison et al., 2016; Clemente et al., 2018; Bourgeois et al., 2019). We detected a significant deviation from a balanced ESR in the populations studied. A strongly male-biased ESR was observed for RJF, Houdan, and Tibetan breeds, whereas the remaining populations exhibited a female-biased ESR. Of these, female-bias was the most obvious in WL and RIR breeds, which are typical commercial chickens. Commercial chickens are often subjected to high-intensity artificial insemination, where many females are inseminated with the semen of a single sire, which greatly increases the proportion of females in the effective population. However, RJF and Tibetan chickens mate naturally; this is not subject to human control, and hence, reflects the true state of the population. It should be noted that sex ratios are in proportion of females that effectively contribute to the gene pool and they should not be interpreted directly in terms of census size. This would provide valuable information on the changes in population dynamics, helping us better understand the unequal contribution of males and females to the gene pool (studying evolution or domestication). In addition, it confirmed that the phenomenon of a female-biased sex ratio in commercial breeds was universal (breeding strategy).

### Sample Data and Numbers

In most diversity studies, it is generally believed that the genetic diversity assessment results are more reliable when the number of individuals in the population is greater than 30. And small sample sizes are enough to assess interpopulation divergence when enough SNP markers are used (Khanlou et al., 2011; Hale et al., 2012; Nazareno et al., 2017). In this study, our populations meet above conditions, so their genetic diversity can represent the genetic diversity level of the entire breed. In addition, for the analysis that may be disturbed by unbalance data, we used random sampling to limit number of individuals and markers, so as to ensure the same level of population analysis, reduce the research error caused by the imbalance of sample number as far as possible.

## Conclusion

In summary, we collected in 1,254 samples representing four different selection pressures. We analyzed the population structure, genetic relationships, genomic inbreeding, ROH, effective population number and ESR using a genome-wide SNPs. Firstly, game breeds were genomically distinct from the other Chinese local chickens. They possessed their own genetic structure in group, and mutual introgression was observed among them. Then, the genetic diversity between commercial and indigenous chickens showed a highly significant different. Commercial chickens with high intense selection had the lowest diversity and local breeds had relatively high diversity. While some local breeds genetic diversity was higher than that of RJF, indicating that the diversity of red jungle fowl was declining in recent years. In addition, ROH level was consistent with estimates of the LD decay trend and effective population size. ROH islands contained many candidate genes controlling phenotypes and production performance. Moreover, there is unequal diversity between sex and autosome chromosomes, and the ESR of commercial breed was distinct from other breeds. Overall, our results provide valuable information to better understanding the genetic diversity and population structure of chicken breeds under different selection pressures.

## Data Availability Statement

The datasets generated for this study can be found in FigShare https://figshare.com/s/efe1bbbf60078f2f08aa.

## Ethics Statement

The animal study was reviewed and approved by the Animal Welfare Committee of China Agricultural University.

## Author Contributions

LQ and ZN conceived and designed this study. JZ performed did the analysis. CN, XLi, and JZ performed the experiments and interpreted the result data. YC, YJ, LW, XLv, and WY contributed to reagents and materials. All authors read and approved the final manuscript.

## Conflict of Interest

The authors declare that the research was conducted in the absence of any commercial or financial relationships that could be construed as a potential conflict of interest.

## Abbreviations

BY: Beijing You
HS: Hongshan
SG: Shouguang
SK: Silkie
TB: Tibet
HD: Houdan
RIR: Rhode Island Red
WL: White Leghorn
Cor: Cornish
LX-G: Luxi gamecock
HN-G: Henan gamecock
XS-G: Xishuangbanna gamecock
ZZ-G: Zhangzhou gamecock
Tur-G: Turfan gamecock
RJF: red junglefowl
PCA: principal component analysis
ESR: effective sex ratio
LD: linkage disequilibrium
ROH: run of homozygosity
Ne: effective population size.
