# Supplementary figures and images for: Genome-Wide Population Genetic Analysis of Commercial, Indigenous, Game, and Wild Chickens Using 600K SNP Microarray Data
Source: Front Genet. 2020 Sep 25;11:543294. doi: 10.3389/fgene.2020.543294 (PMC7545075; doi:10.3389/fgene.2020.543294)

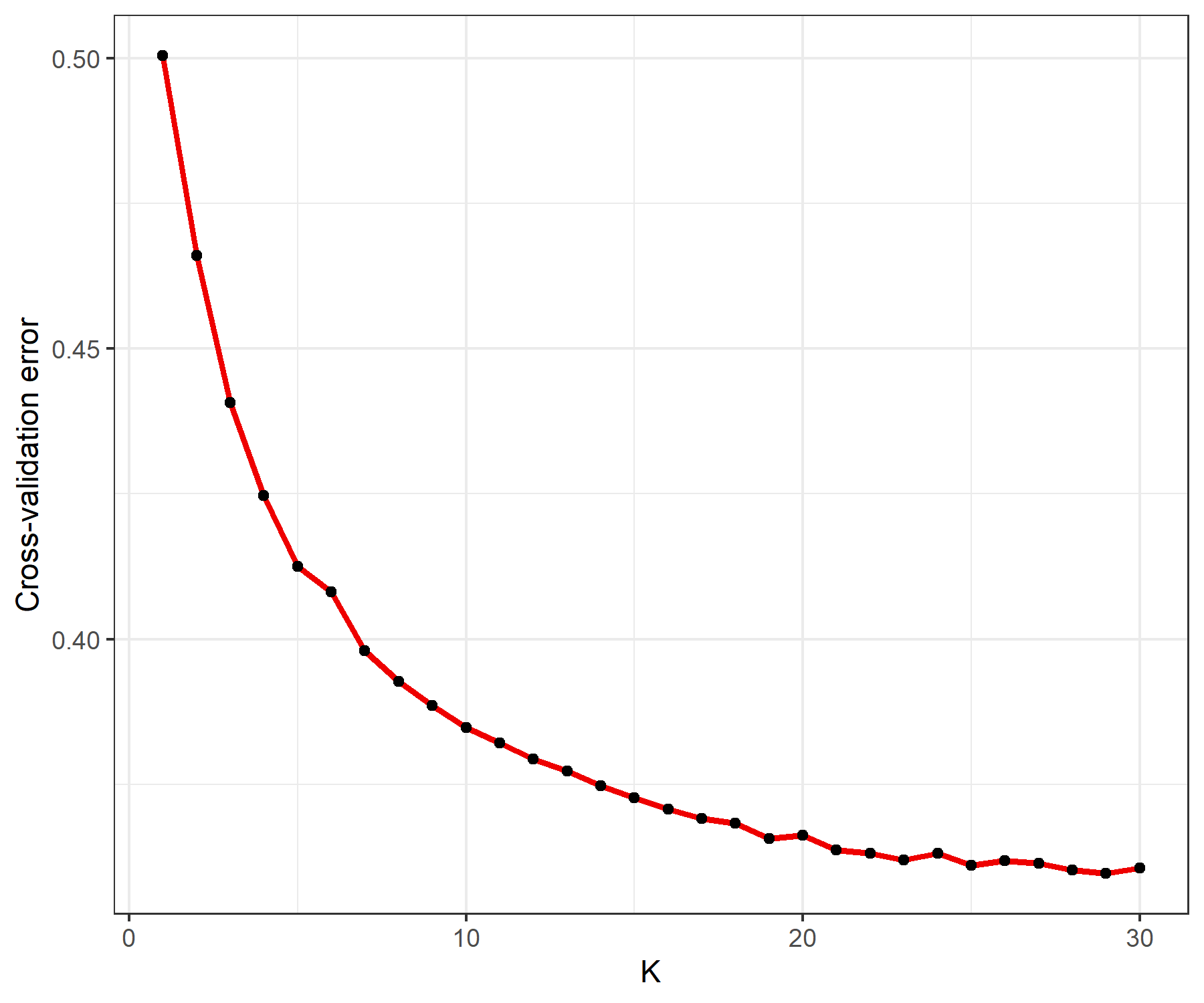

Supplement: Supplementary file 2 [file Image_2.TIFF]
