# Supplementary material for: Genome-Wide Population Genetic Analysis of Commercial, Indigenous, Game, and Wild Chickens Using 600K SNP Microarray Data
Source: Front Genet. 2020 Sep 25;11:543294. doi: 10.3389/fgene.2020.543294 (PMC7545075; doi:10.3389/fgene.2020.543294)
Supplement: Supplementary file 3 [file Table_1.DOCX]

Table S1. The distribution of the SNPs

|  |  | Macro-chromosome | Intermediate-chromosome | Micro-chromosome | Sexual-chromosome | Total |
| --- | --- | --- | --- | --- | --- | --- |
| Before Control |  | 307,493 | 97,608 | 151215 | 26525 | 580,841 |
| After Control | White Leghorn | 209,140 | 68,127 | 95,601 | 12 | 372,880 |
|  | Taihe Silkies | 219,989 | 70,711 | 104,051 | 1,335 | 396,086 |
|  | Shouguang | 204,078 | 66,175 | 97,185 | 1,053 | 368,491 |
|  | Tibetan | 286,290 | 92,352 | 138,529 | 8,100 | 525,271 |
|  | Hongshan | 276,956 | 89,300 | 133,266 | 16,686 | 516,208 |
|  | Beijing You | 239,876 | 77,591 | 113,958 | 16,626 | 448,051 |
|  | Rhode Island Red | 238,046 | 75,381 | 112,436 | 346 | 426,409 |
|  | Houdan | 211,841 | 66,208 | 99,453 | 14,410 | 391,912 |
|  | Red jungle fowl | 171,785 | 55,601 | 81,675 | 14,302 | 323,363 |
|  | Henan Game | 199,029 | 46,532 | 95,028 | 13,307 | 353,896 |
|  | Luxi Game | 235,305 | 76,376 | 112,221 | 17,575 | 441,477 |
|  | Xishuangbanna Game | 248,231 | 80,678 | 120,463 | 19,009 | 468,381 |
|  | Turfan Game | 257,045 | 83,408 | 123,845 | 21,638 | 485,936 |
|  | Zhangzhou Game | 220,400 | 72,643 | 104,194 | 15,768 | 413,005 |
|  | Cornish | 186,558 | 60,072 | 90,970 | 13,771 | 351,371 |

**Note:** Macro-chromosome(1-5), Intermediate-chromosome(6-11), Micro-chromosome(12-28,33), Sexual-chromosome (40,63)
